# Efficacy of using an intravenous catheter to repair damaged expansion lines of endotracheal tubes and laryngeal masks

**DOI:** 10.1186/s12871-022-01776-5

**Published:** 2022-07-26

**Authors:** Tingting Wang, Jiang Wang, Yao Lu, Xuesheng Liu, Shangui Chen

**Affiliations:** 1 Department of Anesthesiology, The First Affiliated Hospital of Anhui Medical University, No.218 Jixi Road, Hefei, 230022 China; 2 Ambulatory Surgery Center, The First Affiliated Hospital of Anhui Medical University, Hefei, 230022 China

**Keywords:** Laryngeal masks, Expansion lines, Endotracheal tube, Anaesthesia, Intensive Care Unit, Intravenous catheter

## Abstract

**Background:**

In perioperative care or intensive care units, the expansion lines of endotracheal tubes (ETTs) or laryngeal mask airways (LMAs) may be accidentally cut off during medical procedures. We designed a simple method for repairing damaged ETT and LMA expansion lines.

**Methods:**

In this *in vitro* study, ETT (*n* = 20) or LMA (*n* = 20) models were each categorized into experimental (*n* = 10) and control (*n* = 10) groups. In the experimental groups, the expansion lines were cut in the middle, and a 22G intravenous catheter was inserted into the broken end of each expansion line. The time taken to repair the expansion lines was recorded in both experimental groups. The repaired expansion lines in both groups were tested for visible underwater air leakage with cuffs under high pressure (120 cm H2O). After 15 h, the cuff pressure and tensile strength of the expansion lines were measured.

**Results:**

The overall time required to repair the expansion line was 27.8 ± 1.5 s in the ETT group and 20.4 ± 1.1 s in the LMA group. When the cuff pressure was increased to 120 cmH_2_O, no air leakage was observed in the experimental LMA and ETT groups. The mean difference in the cuff pressures of the control and experimental groups was insignificant for both, ETT (9.50 ± 1.29 vs. 9.50 ± 1.08 cmH_2_O, 95% CI =  − 1.11 to 1.11 cmH_2_O, *P* = 1.00) and LMA (34.1 ± 1.10 cmH_2_O vs. 34.5 ± 0.97 cmH_2_O, 95% CI =  − 0.57 to 1.37 cmH_2_O, *P* = 0.40) groups, The tensile strength and the force required to pull apart the expansion lines in the experimental groups were lower than those in the control groups for ETTs (3.32 ± 0.37 N vs. 35.03 ± 4.47 N, 95% CI =  − 34.69 to − 28.72 N, *P* < 0.0001) and LMAs (36.55 ± 2.20 N vs. 26.18 ± 1.67 N, 95% CI =  − 12.21 to − 8.53 N, *P* < 0.0001).

**Conclusion:**

An intravenous catheter can be directly inserted into the damaged ETT or LMA expansion lines; it is a simple, rapid, and effective repair method.

**Supplementary Information:**

The online version contains supplementary material available at 10.1186/s12871-022-01776-5.

## Introduction

Establishing artificial airways is an important procedure when administering anesthesia and treating patients in critical condition. Expansion line leakage may endanger the safety of patients in the perioperative period or those in the intensive care unit (ICU) [1, 2]. Underestimating the importance of expansion lines often leads to an emergency situation or can present an obstacle to airway management [3]. Cuff leakage due to breakage of the expansion line is common during perioperative care and in the ICU [4–7]. Previous experience suggests replacing the endotracheal tube (ETT) and performing reintubation; however, the second intubation is highly risky and can cause airway damage, laryngeal oedema, and even death. When the pilot balloon assembly breaks, the best option is to quickly and effectively repair the damaged end. Some studies have reported attempting to repair ruptured or damaged laryngeal mask airway (LMA) or aerated ETT tubes to prevent reintubation [3–8]. However, these methods are inadequate and the acquisition and fabrication of materials can take a long time, which increases the risk to patients. Therefore, we explored the use of easily accessible materials for minimizing delays in patient rescue and simplified the procedures to evaluate the reliability of the proposed repair method.

## Methods

This *in vitro* study was approved by the ethics committee of the First Affiliated Hospital of Anhui Medical University on January 6, 2022 (approval number: PJ2022-01–52). All methods were carried out in accordance with relevant guidelines and regulations. No written informed consent was obtained from participants because it was an *in vitro* abiotic experimental study and did not utilize data from human subjects.

### Experimental and control groups

**Fig. 1:**
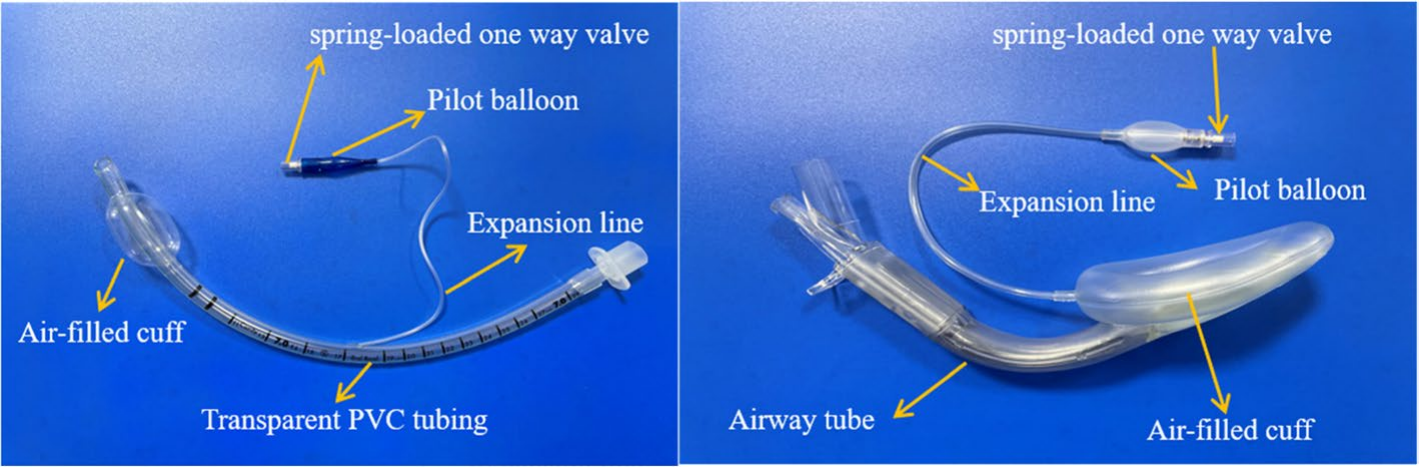
Different components of ETT and LMA. ETT—endotracheal tube; LMA— laryngeal mask airway;

**Fig. 2:**
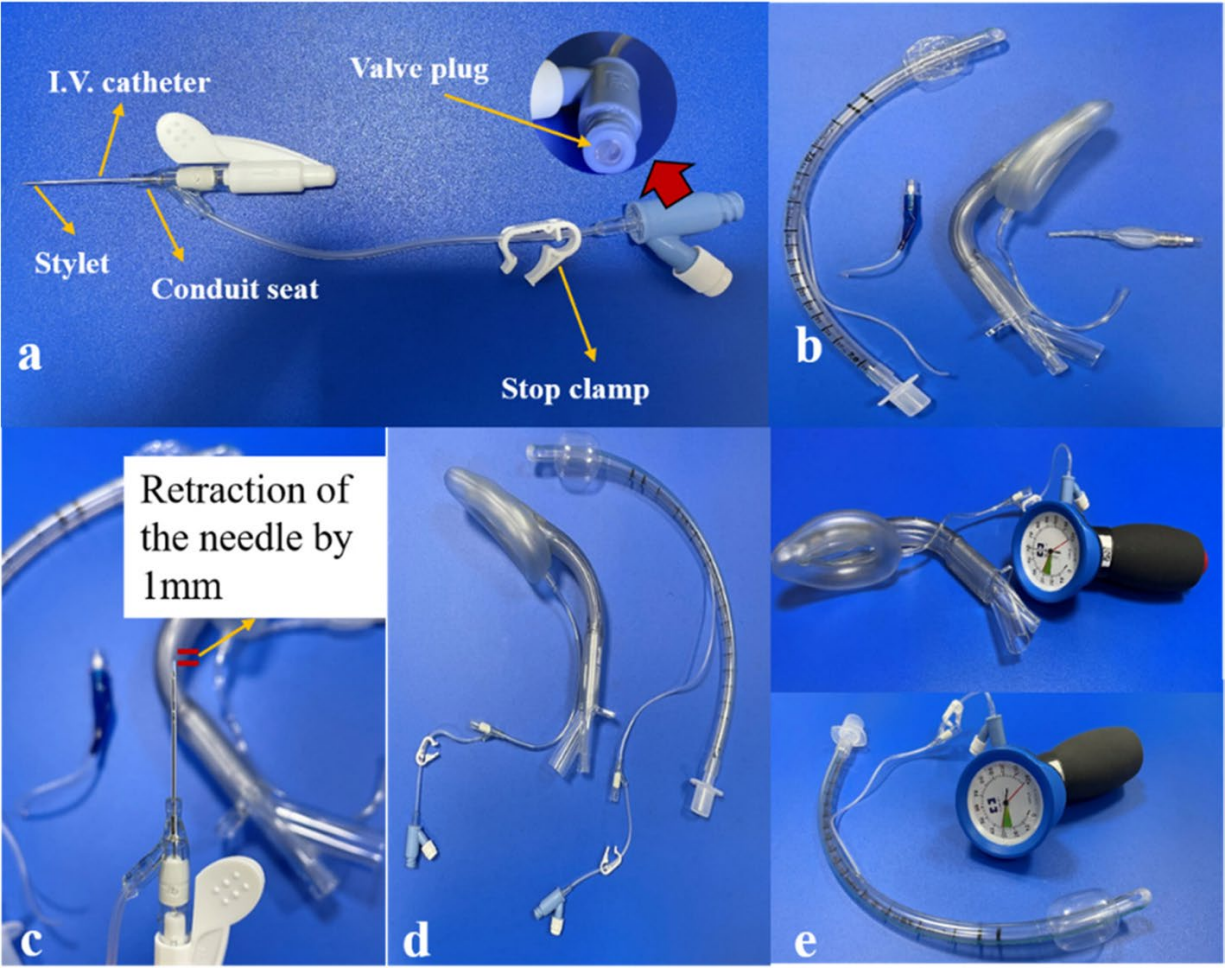
Steps for repairing ETT and LMA expansion lines. Components of a venous indwelling needle (**a**); cutting off the remaining end of the ETT and LMA expansion lines (**b**); retracting the needle by 1 mm prior to its insertion (**c**); connecting it with the remaining end of the airbag expansion lines (**d**); pressure at the ETT and LMA cuff measured using a pressure gauge (**e**). ETT—endotracheal tube; LMA— laryngeal mask airway

The ETT group included 20 sets of ETTs of the same model and size, without production defaults or signs of air leak (*COVIDIEN llc,15 Hampshire Street, Mansfield, MA 02,048 USA, Curity model 7.0*). Of these, 10 ETTs each were assigned to the experimental and control groups. The LMA group included 20 sets of LMAs of the same model and size without production defaults or signs of air leak (LMA supreme model 3, Teleflex Medical, lot 19&1920,Industrial Zone Phase 1, *Kulin Hi-Tech Park, Kulim,09,000 Malaysia*) (Fig. 1). Of these, 10 LMAs each were assigned to the experimental and control groups. A 22G intravenous catheter (*China linhwa Zfii-b type*) was used in both experimental groups. The distal end of the 22G intravenous catheter was inserted into the cut end of the expansion line. Before insertion, the needle was pulled out by 1 mm to restore the expansion line (Fig. 2).

We simulated an *in vitro* tracheal model using a 20-mL plastic syringe in which the plunger was removed (*China jierui 20 ml)*. The internal diameter of the syringe was 2 cm, which is approximately the size of an adult human trachea [9, 10]. In the LMA group, a 35-mm- diameter plastic cylinder with a length of 120 mm was connected to the size 3 LMA to roughly simulate the insertion of LMA into the upper airway [11]. The balloon pressure was measured using VBM Balloon Pressure Gauge Handheld Pressure Pump Tracheal Intubation Pressure Gauge (*German, Hxh-11 Hxh Ce*). The expansion lines in the experimental groups were repaired using an intravenous catheter, whereas those of the control groups were left intact.

### Time required to repair ETT and LMA expansion lines

The repair procedures were performed by a nurse who had never received training on these procedures before, and the time required to repair the expansion lines in the ETT and LMA experimental groups was recorded by an anesthetist. The ETT and LMA expansion lines were cut, and the time from initiation to completion and that required to maintain the cuff at normal pressure were recorded.

### Integrity of the ETT and LMA expansion lines

The integrity of expansion lines were tested underwater after repair using a disposable pressure sensor to monitor the pressure changes of the cuff (*Dominican Republic Edwards PX260*) [12]. In both groups, the balloon pressure of the ETTs or LMAs were adjusted to 120 cmH_2_O. We deliberately used a pressure much higher than that used in a clinical setting (ETT,20–30 cmH_2_O; LMA, 60cmH_2_O) [13–15]. The expansion line models were placed in a full bucket of water, the detector was set to 0 before the measurements were taken, and the measurements were acquired by connecting the pilot balloon valve to an arterial pressure transducer via a three-way stopcock. The pressure was displayed on a physiologic bedside monitor (MINDRAY vs.-600).Leaks were identified through visual observation as bubbles dispersed from the repaired end. We compared the percentage of leaks between the experimental and control groups [16].

### ETT and LMA airbag cuff pressure test

The literature indicates that a small amount of gas is lost when the cuff pressure is measured. We therefore tested the cuff pressure and found that this loss was caused by a small leakage that occurred when the pressure gauge was directly connected to the pilot ball valve and a small amount of air enters the pressure gauge [17]. To avoid this leakage, we clamped the ETT and LMA expansion lines with vascular forceps when the pressure gauge was connected with the balloon to measure the pressure in the control group. In the example groups, we used an intravenous catheter with a stop clamp that was closed when the pressure was measured. This clamp was only used to test the cuff pressure, and it was unnecessary to clamp the expansion line during clinical repair; the stop clamp could be retained or removed as preferred. We adjusted the pressure in the ETT and LAM groups to 30 cmH_2_O and 60 cmH_2_O, respectively;these settings are safe for use in the clinic [18, 19]. After 15 h, we reassessed the pressure of each ETT and LMA using a pressure gauge and compared the pressure changes between the experimental and control groups.

### Tensile strength of the ETT and LMA expansion lines

**Fig. 3:**
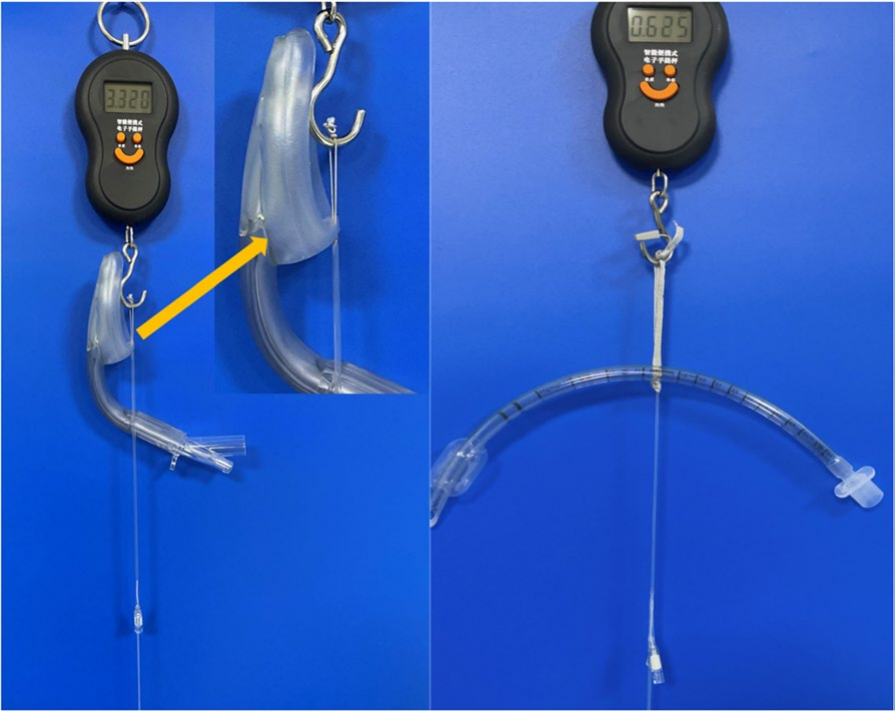
Tensile strength testing of the ETT and LMA inflation tubes. The cotton thread on the ETT and LMA inflation tubes is fixed with an electronic scale and the distal pressure is maintained. ETT—endotracheal tube; LMA— laryngeal mask airway

**Fig. 4:**
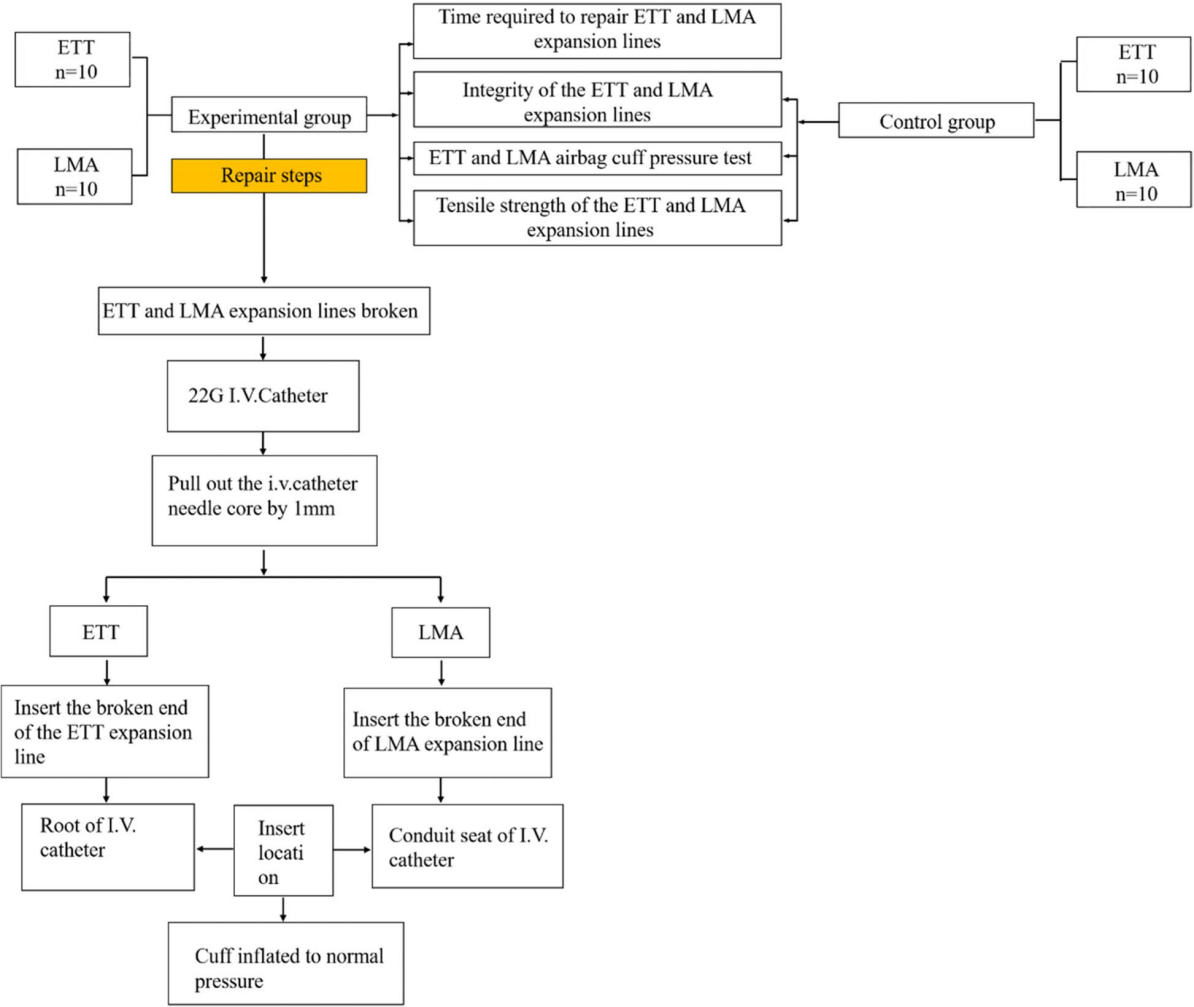
Flowchart for the experimental and control groups. ETT—endotracheal tube; LMA— laryngeal mask airway; I.V. catheter—Intravenous catheter

We tensile strength of the expansion lines was tested in both groups To this end, we used a small and stable electronic scale (Wh-a04, Weiheng, China) with a weighing range of 0.05–50 kg. The end of a cotton thread was fixed on the root of the expansion line of the ETT or LMA, and the other end was fixed on the scale. Then, the electronic scale was hung on a wall, and the end of the intravenous catheter was pulled in the experimental group or the pilot balloon in the control group until the expansion line broke or separated (Fig. 3). The weight on the electronic scale was recorded and converted to Newton (1 kg = 9.8 Newtons). The force required to break or separate the expansion line were compared between the experimental and control groups (Fig. 4).

### Statistical analyses

According to the results of our previous preliminary experiment, the mean ± standard deviation of cuff pressure was 9.4 ± 1.5 cm H_2_O after 15 h in the ETT control group. We believe that the difference of 3 cm H_2_O is clinically significant, and a sample size of 8 in each group was calculated to achieve 80% power and an α value of 0.01. To be conservative, we decided to study 10 ETTs in each group.

After 15 h, the pressure in the LMA control group was 33.6 ± 1.14 cm H2O. The same method used to determine the sample size for the ETT groups was used to calculate the required sample size for the LMA groups, which indicated that a sample size of 8 was required in each group. However, for statistical convenience, 10 LMAs were selected in each group to enable a better comparison of the LMA and ETT groups.

SPSS 22 software was used to perform all statistical analyses. The data are expressed as the means ± standard deviation and confidence intervals (CI). The mean values of the two groups were compared using the t test. Results with a *P* value of < 0.05 was considered statistically significant**.**

## Results

This study consisted of 10 experimental samples of ETTs and LMAs in each group, with 10 control samples. To test the efficacy of intravenous catheter repair of damaged ETT and LMA expansion lines, all expansion lines in the experimental groups were cut off and then repaired by intravenous catheters. The integrity of the expansion lines was tested underwater, showing no air leakage. The repaired assemblies maintained cuff pressure not significantly different from that of intact devices, but the tensile strength of the repaired expansion lines was weaker than that in the control group.

The overall time required to repair the expansion line was 27.8 ± 1.5 s in the ETT group and 20.4 ± 1.1 s in the LMA group (Table 1).

**Table 1:** Time to repair ETT and LMA expansion lines

Number	Time (s)	
	ETT	LMA
1	27	20
2	26	22
3	30	21
4	27	20
5	26	20
6	29	19
7	27	19
8	30	20
9	29	22
10	27	21
*p*-value	0.02047^a^	0.63817^a^
Average value	27.8	20.4
Standard deviation	1.549	1.075

*ETT* Endotracheal tubes, *LMA* Laryngeal mask

^a^T-test

In the experimental group, the pressure used in the ETT and LMA was adjusted to 120 cmH_2_O. A hydrostatic test was performed on each tube; there was no visible air leakage observed in the experimental groups, and the monitor showed no air leakage.

**Fig. 5:**
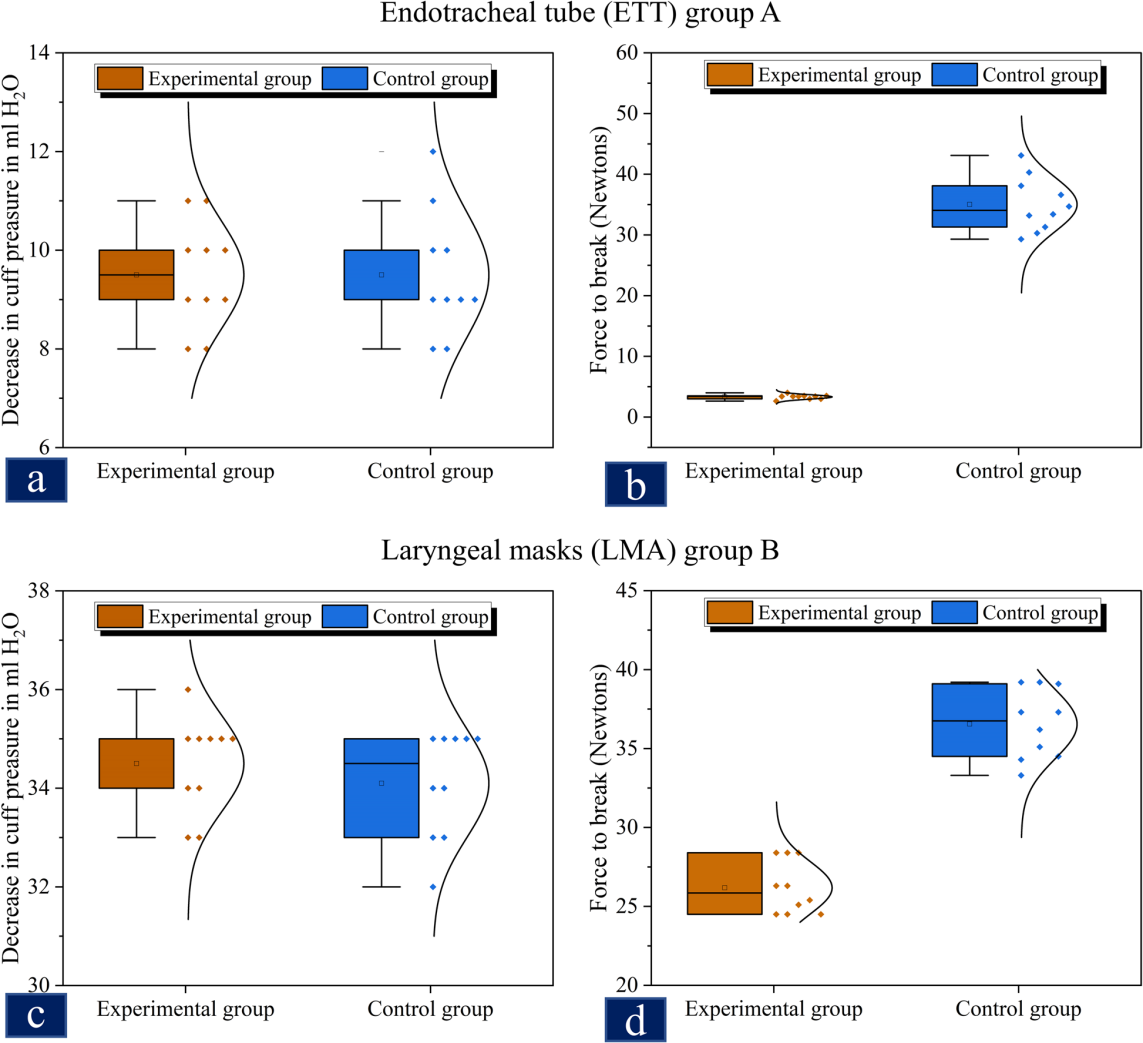
**a** Cuff pressure observed in the ETT group over 15 h. Cuffs from 10 control (blue blocks) and 10 experimental (brown blocks) groups were inflated to 30 cmH20, and tension was measured again after 15 h. There was no significant difference in the pressure decrease from baseline for the two groups (9.50 ± 1.29 vs. 9.50 ± 1.08 cmH_2_O, 95% CI =  − 1.11 to 1.11 cmH_2_O, *P* = 1.00). **b**Force required to break control versus repaired inflation tubes. Segments from control (blue blocks) and experimental (brown blocks) inflation tubes were attached to an electronic scale and pulled down until the tubes broke. The repaired inflation tubes were weaker than the intact lines (3.32 ± 0.37 vs. 35.03 ± 4.47 N, 95% CI =  − 34.69 to − 28.72 N, *P* < 0.0001). **c **Cuff pressures observed in the LMA group over 15 h. Cuffs from the 10 control (blue blocks) and 10 experimental (brown blocks) LMAs were inflated to 30 cmH20, and tension was measured again after 15 h. There was no significant difference in the drop in pressure from baseline between the two groups(34.1 ± 1.10 vs. 34.5 ± 0.97 cmH2O, 95% CI =  − 0.57 to 1.37 cmH2O, *P* = 0.40). **d **Force required to break the control versus repaired inflation tubes. Segments from control (blue blocks) and experimental (brown blocks) inflation tubes were attached to an electronic scale and pulled down until the tubes broke. The repaired inflation tubes were weaker than the control lines (36.55 ± 2.20 vs. 26.18 ± 1.67 N, 95% CI =  − 12.21 to − 8.53 N, *P* < 0.0001). ETT—endotracheal tube; LMA— laryngeal mask airway; CI—confidence interval; N—Newton

The ETT group comprised 10 intact and repaired ETTs each, and pressure measurements were conducted for all. After 15 h, there was no significant difference between the pressures in the two groups (experimental vs. control, 9.50 ± 1.29 vs. 9.50 ± 1.08 cmH_2_O, 95% CI =  − 1.11 to 1.11 cmH_2_O, *P* = 1.00, Fig. 5a. The tensile strength of the repaired expansion lines in the ETT experimental group was lower than that in the control group (3.32 ± 0.37 vs.35.03 ± 4.47 N, 95% CI =  − 34.69 to − 28.72 N, *P* < 0.0001; Fig. 5b).

The LMA group comprised 10 intact and repaired LMAs each, and pressure was measured for all. After 15 h, there was no significant difference between the experimental and control LMAs (34.1 ± 1.10 vs. 34.5 ± 0.97 cmH_2_O, 95% CI =  − 0.57 to 1.37 cmH_2_O, *P* = 0.40; Fig. 5c). The tensile strength of the repaired expansion lines in the LMA experimental group was lower than that in the control group (36.55 ± 2.20 vs.26.18 ± 1.67 N, 95% CI =  − 12.21 to − 8.53 N, *P* < 0.0001; Fig. 5d).

## Discussion

From the currently available evidence, the method of repair developed in this study affords extremely high pressure in ETT and LMA cuffs; however, we found that the tensile strength observed in the ETT and LMA experimental groups was lower than that observed in the control groups. Therefore, medical operations should be performed with high caution, and we recommend the use of adhesive tape at the fracture site to enable repair end realism. Furthermore, if the pilot balloon breaks or the valve is faulty, we can cut the expansion lines. Patching was performed using our described method. To the best of our knowledge, ours study is the first to prove that using an intravenous catheter in the clinic allows the simultaneous repair of two artificial airway devices, ETT and LMA. Our proposed method has distinct advantages over the previously reported patching method [6, 20].

The establishment of an artificial airway is an important component of treating critically ill patients. In perioperative care or the ICU, it is necessary to manage the airways and pilot balloons to ensure the patient’s safety. During administration of anaesthesia during perioperative care or in the ICU, the common causes of cuff rupture are bite, tears, accidental cutting of the expansion line, or failure of pilot balloon [21–23]. If the treatment is not timely, oral secretions and gastric contents can enter the airway, leading to aspiration pneumonia, which can result in death in severe cases [24]. It has been reported that a broken inflation tube can be clamped with a syringe air supply vessel clamp to ensure adequate airbag pressure [25]; however, this method prevents the measurement of balloon pressure. For instance, excessive inflation of the endotracheal tube may cause the pressure in the pilot balloon to increase exponentially, leading to an increased risk of serious injury, including tracheal mucosal ischemia, ulcer, necrosis, tracheoesophageal fistula, and even tracheal rupture. Excessive injection of the LMA will also increase the cuff pressure, resulting in pharyngeal mucosal compression and even ischemic necrosis. Postoperative complications, such as severe pharyngeal pain, eating difficulty, and hoarseness, may occur [26–28]. Several previous studies have described eight catheter balloon repair methods. Whiteside et al. used a syringe to directly clamp the blood vessel clamp after gas injection in the inflation tubes to restore the airbag pressure [29]. Barrion et al. proposed that a closing cap can be used after clamping the blood vessel clamp to maintain the airbag pressure [30]. However, these methods do not allow the cuff pressure to be monitored. Yoon et al. used a metal puncture needle, intercepted the middle needle stem, and inserted both ends of the needle stem into the two sides of the expansion lines to maintain the airbag pressure [31]. However, there are several disadvantages to this method. First, improper cutting of the needle can completely block the needle tip or narrow lumen, preventing the air from entering the airbag. Second, if the cutting end is sharp, it is easy to puncture the connecting tube when joining the connecting lines. The process of attaching the needle stem to the stump is complex because the material is small. This factors results in increased risk for the patient. Additionally, this device cannot be used during magnetic resonance imaging. Furthermore, emergency repair of endotracheal tube balloons takes a long time and increases the amount of work performed by healthcare workers. Dayan et al. used a puncture needle to repair the line by connecting both ends to maintain balloon pressure [16]. Due to the lack of tube core support, it is difficult to connect the two ends during the operation. Furthermore, the material is small, and improper usage can be dangerous to patients. Chaudhuri et al. described the use of an epidural puncture needle to directly connect the other end of the ETT balloon expansion lines. Although it is effective, the materials required for this repair method are not easy to obtain in an ICU [8]. Owusu et al. described the use of a venous catheter for connecting the end of a cut-off expansion line. When measuring the balloon pressure, it is necessary to use the three-way valve [32]. Hills et al*.* described the use of a central venous catheter to repair the ruptured expansion line and strengthen the integrity of the LMA; however, central venous catheters are costly [5]. Singh et al. described a method of reconstructing theexpansion lines. As with the above method, an additional T-tube is required to achieve the complete use function of LMAs [20]. When the ETT and LMA expansion line is broken in a clinical setting, it should be quickly optimized at the fracture ends to prevent reintubation. Although the above studies repaired the LMA or ETT without reintubation, it took a long time to obtain materials, which increased the potential risk to patients. Our study found that using 22G intravenous catheter to repair the broken expansion line of ETT or LMA has the advantages of being simple and requiring materials that are easy to acquire unlike the existing methods. In addition, this procedure only requires a venous indwelling needle; the end of the intravenous indwelling needle that is used for liquid injection provides a valve plug, which is safe and sealed. A three-way valve is not required, and no air leakage will occur. Moreover, this procedure allows the accurate measurement of the balloon pressure even in an intense magnetic field. Simultaneously, the results show that the venous catheter can be used to repair both the ETT and LMA, effectively prevent air leakage, and save the pressure in the expansion line.

This method still has several limitations. First, the connecting line of an endotracheal tube can be too deep and difficult to access. Second, the number of types of ETTs and LMAs used for adults and children in our department is limited. Products from other manufacturers may function differently after undergoing this repair method. Finally, cuff pressure testing was performed using an *in vitro* model. Although our experimental conditions are similar to those used in a clinical setting, the procedure used in this study to measure pressure may be different from that used in human patients.

## Conclusions

Our study indicates that intravenous catheters can be used to repair failed and fractured pilot balloon components in ETT or LMA and that the normal cuff pressure for ETT and LMA can be maintained after repair. This method can be used in a clinical setting and will help shorten the rescue time of patients, prevent reintubation, and ensure the patients safety.

## Supplementary Information

**Additional file 1.** 

**Additional file 2.** 

**Additional file 3.**

## Data Availability

The datasets analysed during the current study are available from the corresponding author upon reasonable request.

## Abbreviations

ICU: Intensive Care Unit
ETT: Endotracheal tubes
LMA: Laryngeal mask
N: Newton
