# Supplementary material for: Efficacy of using an intravenous catheter to repair damaged expansion lines of endotracheal tubes and laryngeal masks
Source: BMC Anesthesiol. 2022 Jul 26;22:238. doi: 10.1186/s12871-022-01776-5 (PMC9316419; doi:10.1186/s12871-022-01776-5)
Supplement: Supplementary file 1 — Additional file 1. [file 12871_2022_1776_MOESM1_ESM.docx]

Catheter Size Required for Repair of different Expansion Lines

| Endotracheal tube / laryngeal mask manufacturer | Internal Diameter (mm) | Catheter Size for Repair(gauge) |
| --- | --- | --- |
| LMA supreme | 3,4 | 18、20、22、24、26 |
| Intersurgical | 2.0,2.5 | 22 |
| COVIDIEN Curity | 4.5,5.5-8.0 | 22 |
| COVIDIEN Shiley | 3.5,4.0,5.0-7.5,35,37,39 | 22 |

The size of conduit seats in different intravenous catheters are all the same, so the intravenous catheter can be used to repair different expansion lines of ETT and LMA.

ETT—Endotracheal tubes; LMA—Laryngeal mask
