# Supplementary figures and images for: Efficacy of using an intravenous catheter to repair damaged expansion lines of endotracheal tubes and laryngeal masks
Source: BMC Anesthesiol. 2022 Jul 26;22:238. doi: 10.1186/s12871-022-01776-5 (PMC9316419; doi:10.1186/s12871-022-01776-5)

**
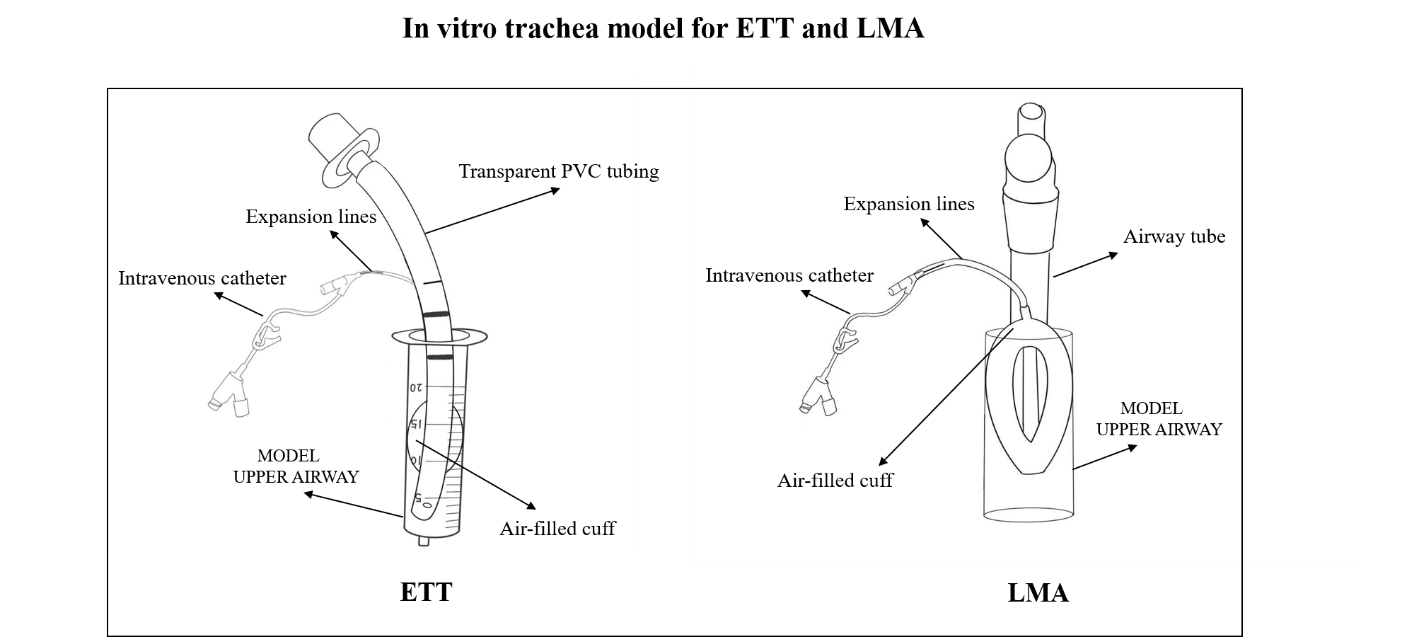
In vitro trachea model for ETT and LMA**

Supplement: Supplementary file 2 — Additional file 2. [file 12871_2022_1776_MOESM2_ESM.docx]
