# Supplementary material for: Efficacy of using an intravenous catheter to repair damaged expansion lines of endotracheal tubes and laryngeal masks
Source: BMC Anesthesiol. 2022 Jul 26;22:238. doi: 10.1186/s12871-022-01776-5 (PMC9316419; doi:10.1186/s12871-022-01776-5)
Supplement: Supplementary file 3 — Additional file 3. [file 12871_2022_1776_MOESM3_ESM.doc]

Supplementary figures.


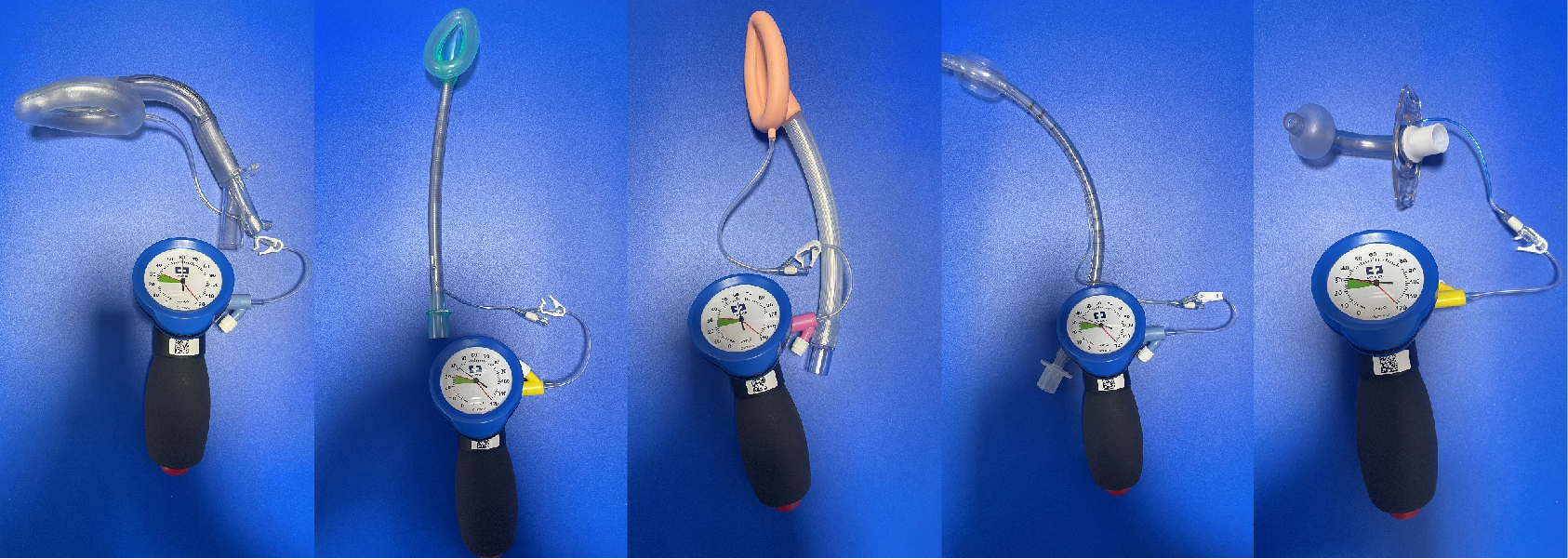


Our in vitro experiment shown that, the method also can be used to repair damage in various pilot balloon assemblies.
